# Apnea-hypopnea index estimation using overnight chest-wall accelerometry

**DOI:** 10.3389/frsle.2026.1858267

**Published:** 2026-07-20

**Authors:** Fons Schipper, Pedro Fonseca, Angela Grassi, Marco Ross, Fokke B. van Meulen, Merel van Gilst, Pien F. N. Bosschieter, Emily Schoustra, Ruud J. G. van Sloun, Fayçal Abdenbi, Nico de Vries, Raphael Heinzer, Jean-Louis Pépin, Sebastiaan Overeem

**Affiliations:** 1 Philips Sleep and Respiratory Care, Eindhoven, Netherlands; 2 Department of Electrical Engineering, Eindhoven University of Technology, Eindhoven, Netherlands; 3 The Siesta Group, Vienna, Austria; 4 Center for Sleep Medicine Kempenhaeghe, Heeze, Netherlands; 5 Department of Otorhinolaryngology, Head and Neck Surgery OLVG West, Amsterdam, Netherlands; 6 Centre d'investigation et de recherche sur le sommeil, Lausanne, Switzerland; 7 Université Grenoble Alpes, Grenoble, France

**Keywords:** accelerometry, obstructive sleep apnea, positional sleep apnea, sleep, sleep monitoring, sleep position therapy, sleep staging, transfer learning

## Abstract

**Rationale and study objectives:**

To develop a method for apnea-hypopnea index (AHI) estimation using a chest-worn accelerometer, as an approach to obstructive sleep apnea diagnosis and long-term monitoring of treatment, for example through positional therapy.

**Methods:**

We developed a method for AHI estimation by combining a cardiorespiratory sleep staging algorithm with an adapted neural network for detecting respiratory events. Originally based on electrocardiography and respiratory impedance plethysmography, the network was retrained using chest-wall accelerometry-based instantaneous heart rate and respiratory effort. Training and validation utilized accelerometer data from 413 participants across two centers, recorded during diagnostic overnight polysomnography (PSG), in absence of any therapy. The dataset was split equally: half was used to train the neural network, and the remaining half to evaluate its performance. This evaluation compared the accelerometry-derived overnight AHI against the reference from PSG, both overall and separately for supine and non-supine sleeping positions.

**Results:**

Sleep staging reached substantial agreement with polysomnography, achieving a Cohen's kappa coefficient of agreement of 0.67 for four-class sleep staging (Wake/REM/N1-N2/N3). AHI was estimated with highly reliable, showing an intraclass correlation coefficient of 0.90 (95% CI: 0.86–0.92) when compared to polysomnography-derived values. Performances were consistent in both supine and non-supine sleeping positions. Positive likelihood ratios were high (7.1, 16.0, and 165.7 for the mild, moderate, and severe OSA severity classes, using near-boundary double labeling). Negative likelihood ratios were low (0.095, 0.153, and 0.069 for the same cases).

**Conclusion:**

AHI can be reliably estimated using chest-wall accelerometry. This approach may be used in diagnostic tests for OSA but also to assess residual AHI during positional therapy.

## 1 Introduction

Untreated obstructive sleep apnea (OSA) and its associated poor sleep quality can adversely impact short-term well being and long-term health (Kendzerska et al., 2014; Morsy et al., 2019). The management of OSA can benefit from continuous monitoring during sleep, focusing on the apnea hypopnea index (AHI) which can be used as an indicator of disease severity and as a measurement of therapy efficacy (International Classification of Sleep Disorders, 2023; Pevernagie et al., 2020).

A commonly used OSA therapy is positive airway pressure (PAP). Modern PAP devices track the AHI on a nightly basis by measuring apnea and hypopnea events derived from airflow (Iftikhar and BaHammam, 2023). Given the challenges with PAP therapy compliance, alternative therapies such as positional therapy (PT) have been developed. PT treats positional obstructive sleep apnea (pOSA), a condition where patients have a significantly higher OSA severity in the supine position, and a much lower severity in other sleeping positions (Oksenberg et al., 2020). High prevalence rates of pOSA have been reported, with over 50% of patients referred for OSA evaluation presenting this phenotype (Heinzer et al., 2018). PT devices continuously monitor body position and provide feedback (usually with vibrations) when in supine position, triggering body position changes (Ravesloot, 2024). Although having demonstrated compliance and efficacy (Berry et al., 2019), PT devices do not measure airflow and consequently cannot monitor AHI and provide a comprehensive assessment of therapy efficacy. PT devices are typically mounted on the chest wall and incorporate an accelerometer to monitor body position.

Body-worn inertial sensors (i.e., accelerometers and gyroscopes) have long been used in sleep research and in clinical practice. The most established technique, actigraphy, measures gross body movements to differentiate between sleep and wakefulness (Sadeh et al., 1995). While effective for basic sleep detection, actigraphy often misidentifies wakefulness as sleep during periods of motionlessness (Gao et al., 2022).

Researchers have explored the placement of sensors in proximity to the airways to potentially capture respiratory mechanics. Morillo et al. (2010) demonstrated that a piezoelectric accelerometer on the suprasternal notch could capture cardiac, respiratory and snoring activity (Morillo et al., 2010). Pepin et al. (2022) explored the measurement of mandibular jaw movements with inertial sensors and showed how these correlate with respiratory effort and in turn, can be used to accurately estimate the AHI (Pepin et al., 2022).

Parallel efforts have focused on measurements on the chest wall. One study used a chest-mounted 3D accelerometer to distinguish sleep and wake by combining activity and sleep position data (Zjouyan et al., 2017). Another study introduced a “Chest Effort Index” calculated from the peak-to-trough exertion heights of respiratory movements (Hsu et al., 2020). Ryser et al. (2022) developed manually engineered features describing time- and frequency-domain characteristics of the filtered magnitude of chest-wall movements and used machine learning to distinguish “disrupted” or “regular” breathing in a small cohort (Ryser et al., 2022).

However, to the best of our knowledge, no studies attempted to estimate the AHI from chest-worn inertial sensing. In this work, we developed and validated such an approach, based on a model that uses continuous respiratory effort (RE) and instantaneous heart rate (IHR) as inputs, solely derived from chest-worn accelerometry. We employed a transfer learning approach to adapt an RE/IHR based model, which originally relied on RE from respiratory belts and IHR from ECG. Performance was then assessed against the AHI from gold-standard PSG.

In this study, we investigated if the AHI can be estimated reliably from a chest-worn accelerometer. To this end, an accelerometer was worn during a diagnostic sleep study using PSG, in the absence of therapy. A neural network model for respiratory event detection was trained on half of the dataset, while the remaining half was reserved for evaluation. Performance was assessed by comparing the estimated overnight AHI from chest-wall accelerometry against the AHI derived from PSG. We also evaluated the effect of position (supine vs. non-supine) on performance.

## 2 Methods

We adapted a previously developed neural network designed for respiratory event detection, which utilized ECG and Respiratory Inductance Plethysmography (RIP) belt data, to accept inputs derived entirely from chest-wall accelerometry. The adaptation was performed using transfer learning due to the limited availability of simultaneous recordings featuring both Polysomnography (PSG) and chest-wall accelerometry. Because the Apnea-Hypopnea Index (AHI) calculation requires Total Sleep Time (TST), we first evaluate the performance of the previously established accelerometer-based sleep staging method on the present dataset. Finally, we assessed the method's suitability for direct implementation in Positional Therapy (PT) devices by evaluating its performance separately for supine and non-supine body positions.

### 2.1 Dataset

The dataset comprised 413 subjects referred for overnight PSG as part of their standard clinical procedure. Data were collected from two distinct clinical sites. In the first site, the OLVG hospital (Amsterdam, the Netherlands) data were collected between June 3, 2021, and December 12, 2022, among 124 adults scheduled for ambulatory PSG. The study was reviewed and approved by the local scientific advice committee (Adviescommissie Wetenschappelijk Onderzoek, ACWO) of the OLVG hospital (File no: WO 20.134). In the second site (Sleep Medicine Center Kempenhaeghe Heeze, the Netherlands), data were collected between January 27, 2020, and November 11, 2022, and comprised 281 adults referred for overnight in-lab diagnostic studies, as part of the Sleep and OSA Monitoring with Non-Invasive Applications (SOMNIA) study (Van Gilst et al., 2019). The SOMNIA study was reviewed by the medical ethical committee of the Maxima Medical Center (Veldhoven, the Netherlands; file no: N16.074 and W17.128).

**Figure 1:**
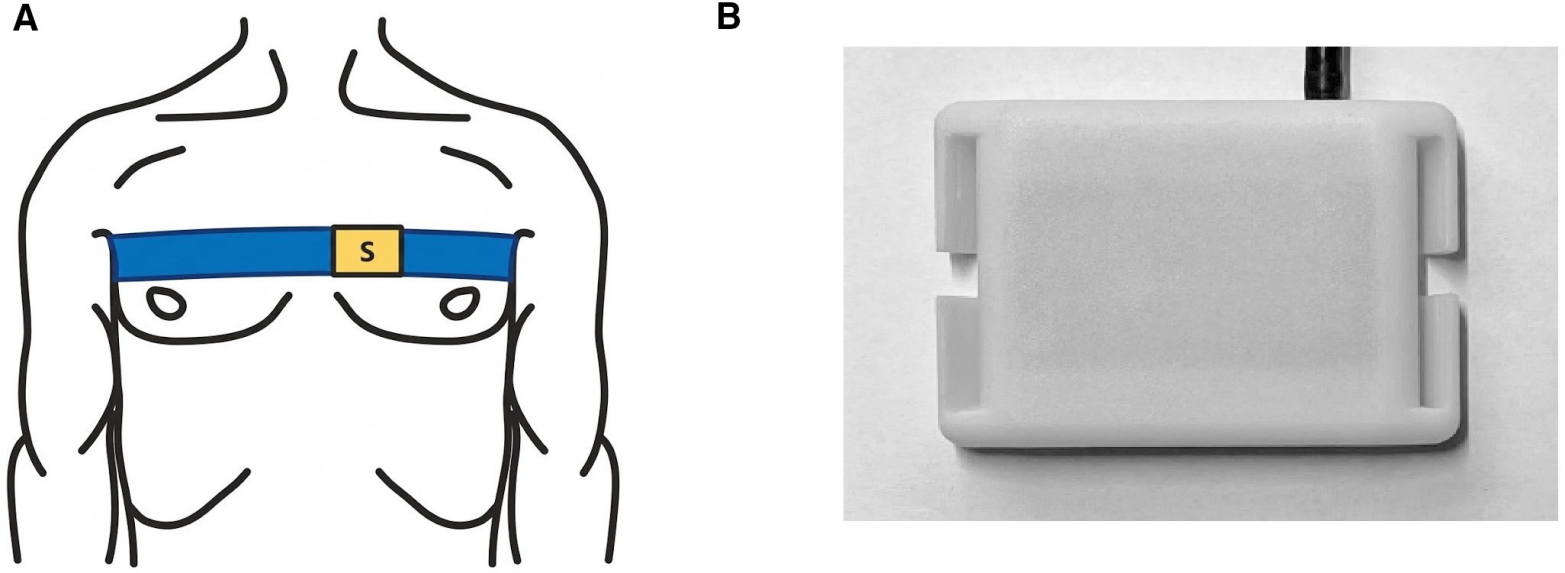
**(A)** Location of the sensor device (S) on the thoracic belt of the participant. **(B)** The 3D printed enclosure of the device including the accelerometer, measuring 62 × 48 × 14 mm and weighing 30 grams, with the cable to a separate data logger, on the top-right. A later version of the device had the same outline but no cable, with data logging performed in the actual unit.

In addition to the standard PSG setup at both sites, participants wore a single 3D accelerometer (ADXL355, Analog Devices, Wilmington MA, USA) mounted on the thoracic Respiratory Inductance Plethysmography (RIP) belt, see Figure 1. This sensor was configured to acquire triaxial raw accelerometer data at 250 Hz.

All participants gave informed consent before participating. The studies met the ethical principles of the Declaration of Helsinki, the guidelines of Good Clinical Practice and the current legal requirements. The protocols for the data analysis described in the present study were approved by the local scientific advice committee of the OLVG hospital, by the review boards of the Medical Ethical Committee of the Kempenhaeghe hospital and by the Internal Committee of Biomedical Experiments of Philips Research.

To ensure standardized scoring of sleep stages and sleep disordered breathing (SDB) events (specifically, apneas and hypopneas) across the recordings of both sites, the data were automatically scored using Somnolyzer 4.1 (Philips, Monroeville, PA, USA; Anderer et al., 2022). Somnolyzer has been validated against human scoring following the American Academy of Sleep Medicine (AASM) rules for scoring of sleep stages, SDB events and cortical arousals and was certified by the AASM through their Autoscoring Certification Program (Celmer, 2024). Somnolyzer was configured to score events using the current recommended rules (Troester et al., 2023): apneas were defined as a (near-)complete cessation of airflow (at least 90% reduction from baseline), for at least 10 s; hypopneas were scored based on a reduction of airflow between 30 and 90% from baseline, lasting at least 10 s, and associated with an oxygen desaturation of at least 3% or a cortical arousal.

### 2.2 Data preparation

Precise synchronization between the Polysomnography (PSG) and accelerometer recordings was essential for comparing the derived sleep stages and events, but the internal clocks of the independent acquisition devices were not aligned. Therefore, an intrinsic synchronization method was required.

The accelerometer data were first processed to obtain the precise interbeat intervals (IBIs) and temporal locations for each heartbeat. IBIs were detected by exploiting their local periodicity and were combined with peaks in the acceleration signal to accurately localize the heartbeats in time (Schipper et al., 2024b). IBIs and temporal locations were derived from the PSG as well. R-peaks were localized in the ECG signal by means of a non-linear transformation in combination with first-order Gaussian differentiation (Kathirvel et al., 2011). IBIs were subsequently derived by computing the time differences between the localization times of the R-peaks. Synchronization was finally achieved by using the PSG clock as reference, and by adjusting the rate and offset of the clock of the acceleration signal such that the cross-correlation between the two IBI time series was maximized.

### 2.3 Data processing

**Figure 2:**
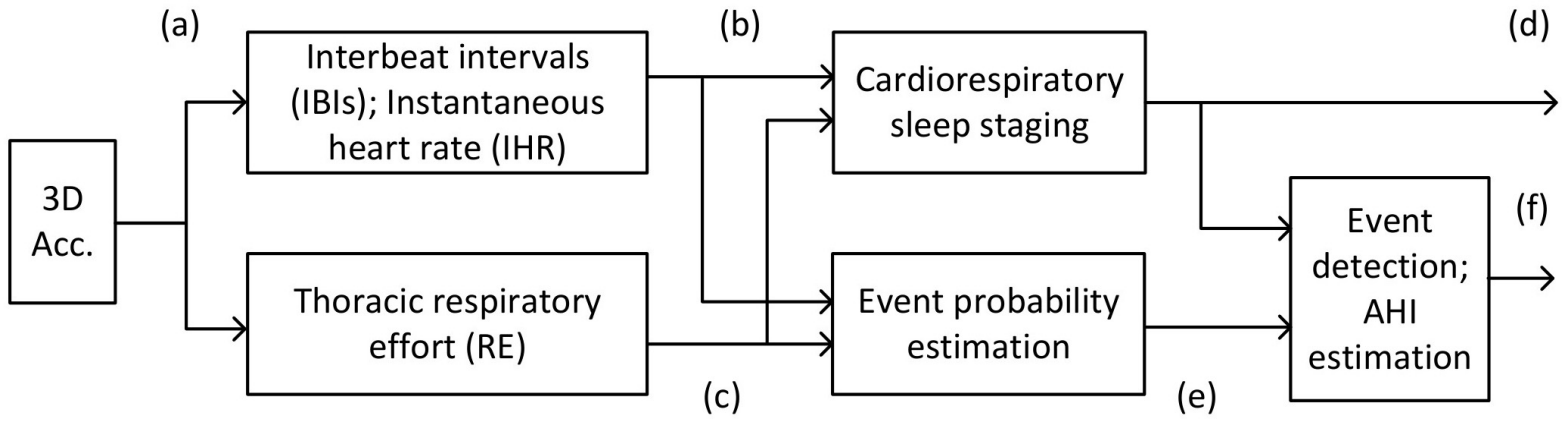
Data processing block diagram. The labels **(a–f)** refer to the example waveforms in this Figures 3 and 4. Using the 3D acceleration **(a)**, the IHR is estimated from IBIs **(b)**. Estimated RE **(c)** is used for cardiorespiratory sleep staging **(d)** and estimation of probabilities of apnea and hypopnea events **(e)**. Finally, using sleep stage-dependent thresholds, events are detected, counted, and divided by the total sleep time to obtain an estimate for the AHI **(f)**.

**Figure 3:**
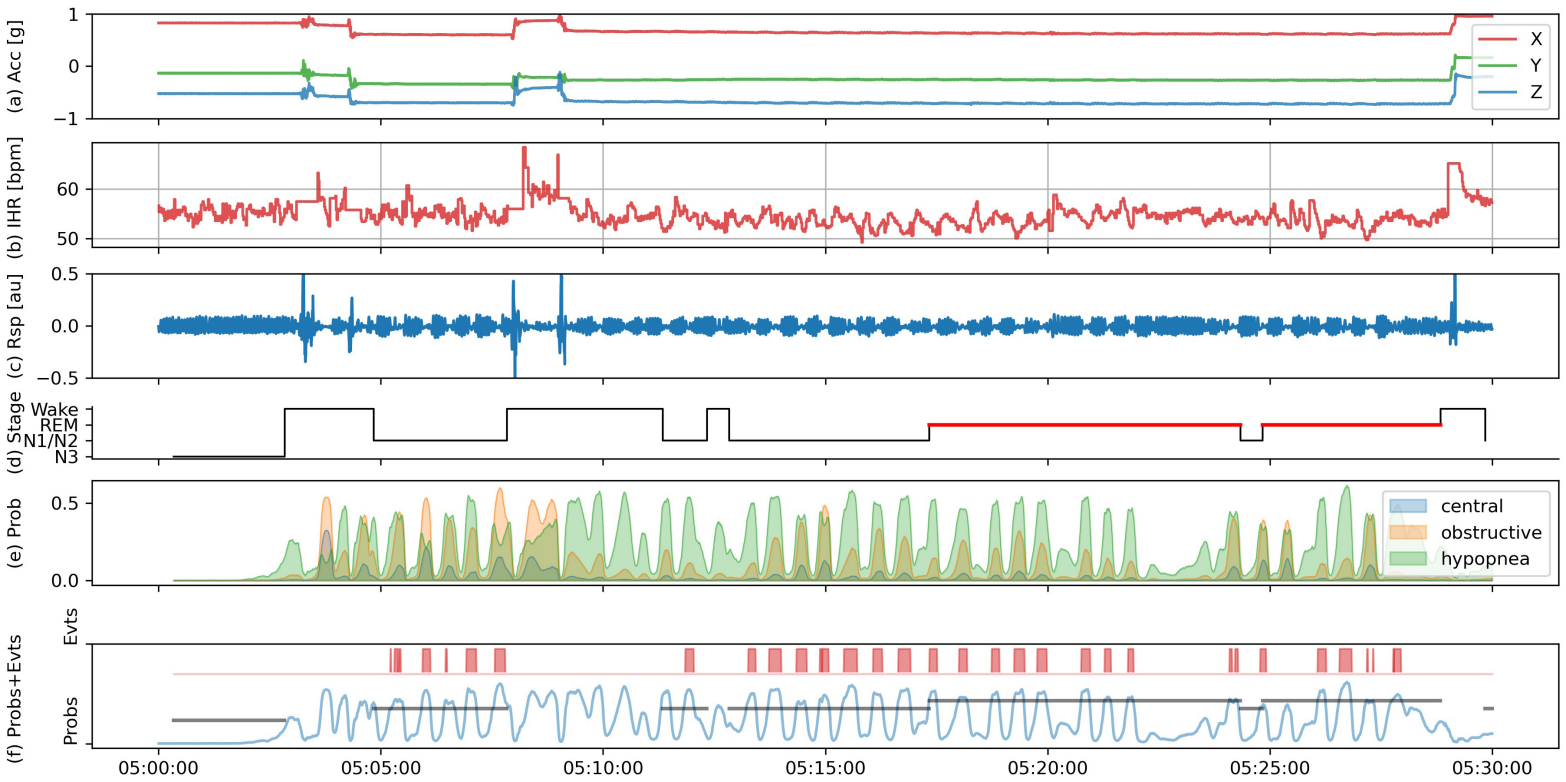
Example waveforms. The labels **(a–f)** refer to outputs of processing blocks in Figure 1: **(a)** 3D acceleration from the accelerometer, **(b)** instantaneous heart rate, **(c)** respiratory effort, **(d)** sleep stages estimated from the instantaneous heart rate (IHR) and respiratory effort (RE), **(e)** SDB event probabilities estimated from instantaneous heart rate and respiratory effort, **(f)** combined SDB event probabilities with sleep stage-dependent thresholds.

**Figure 4:**
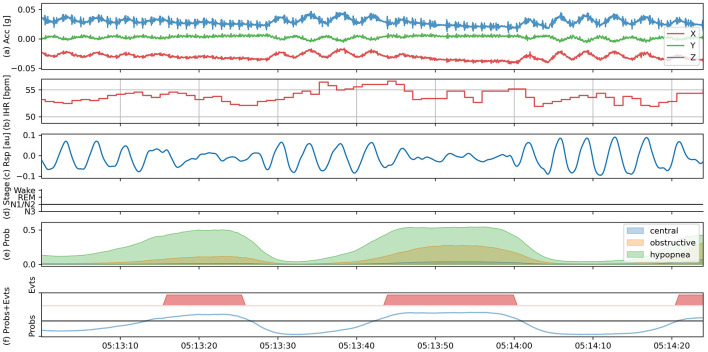
Example waveforms—zoomed in to a 1.5-min interval. The labels **(a–f)** refer to outputs of processing blocks in Figure 1: **(a)** 3D acceleration from the accelerometer, **(b)** instantaneous heart rate, **(c)** respiratory effort, **(d)** sleep stages estimated from the instantaneous heart rate (IHR) and respiratory effort (RE), **(e)** SDB event probabilities estimated from instantaneous heart rate and respiratory effort, **(f)** combined SDB event probabilities with sleep stage-dependent thresholds.

All steps required to estimate sleep stages and AHI from the accelerometer signal are illustrated in Figure 2 and further described in detail in the following sections. The labels **(a)** to **(f)** refer to Figure 2 (block diagram), Figure 3 (example waveforms), and Figure 4 (zoomed-in waveforms).

#### 2.3.1 Cardiorespiratory measurements

The previously developed neural network for respiratory event detection (Fonseca et al., 2024) relied on respiratory effort (RE) and instantaneous heart rate (IHR). Originally, RE was derived from a respiratory belt, while IHR was derived from ECG. Here, we derive RE and IHR solely from a chest-worn accelerometer using the methods described in detail in previous publications (Schipper et al., 2023, 2024b) and summarized here. Figures 3, 4 illustrate examples of RE and IHR signals. The slow components of the accelerometer signals, visible in Figure 4a, are caused by respiration. The RE signal (Figure 4c) was estimated from these signals using a pre-trained convolutional neural network. This network modeled the complex relationship between the triaxial accelerometer signal (representing chest-wall movements) and the reference RIP signal used for training. The output of the network was a 10 Hz time series intended to represent the RE signal, mimicking the morphology of a reference RIP signal. The small spikes, mainly visible in one of the accelerometer axes (in blue) in Figure 4a, are caused by heartbeats. The IHR signal (Figure 4b) was derived from the accelerometer data exploiting the principles of Seismocardiography. IBIs were estimated by exploiting the local periodicity in the accelerometry signal and heartbeats were localized in time. The sequence of localization times and IBIs was converted into IHR by taking the reciprocal of IBIs. Finally, the IHR time series was uniformly sampled at 10 Hz using a zero-order hold interpolation.

To assess the quality of the cardiorespiratory measurements used in this study, we evaluated the performance of accelerometer-derived RE and IBIs against gold-standard measurements. We then compared these results to the performance reported in previous study cohorts ACC-RE-72 (Schipper et al., 2023) and ACC-IBI-147 (Schipper et al., 2024b), of which details may be found in the Supplementary material.

#### 2.3.2 Cardiorespiratory sleep staging

The estimation of AHI requires an estimation of TST, which in turn requires a classification of the epochs during which the participant is awake or asleep. Furthermore, as the salience of respiratory events in terms of their cardiorespiratory expression depended on the sleep stage during which they occurred, the detection of respiratory events also required sleep stages (see Figure 2).

As we did not want to retrain a previously developed sleep stage classifier and as we needed the sleep stages for event detection, we used a previously developed algorithm for cardiorespiratory sleep staging (CReSS; Bakker et al., 2021). The CReSS algorithm was originally developed to perform four-class sleep staging (Wake, N1-N2, N3, and REM) using cardiorespiratory signals. We previously demonstrated that CReSS could be successfully used to perform the same four-class staging using only RE and IHR derived from chest-wall accelerometry (Schipper et al., 2024a). Using that algorithm, TST was estimated simply by counting the time spent in any of the detected sleep stages (except Wake).

#### 2.3.3 Event detection and AHI estimation

Apnea and hypopnea events (henceforth referred to simply as SDB events) were detected using a neural network previously developed to detect these from IHR derived from ECG and RE derived from a RIP band (Fonseca et al., 2024). The original neural network was trained with over 2,000 recordings and further validated on more than 600 additional recordings. Such amounts of data are not available for PSG recordings with concurrent chest-wall accelerometry; hence, we leverage transfer learning, whereby a neural network trained on a larger, more readily available dataset, is fine-tuned using a smaller target-specific dataset. This technique has been applied successfully in the domains of ECG (Gliner et al., 2023), PPG (Davies et al., 2023), and sleep (Waters and Clifford, 2022).

**Figure 5:**
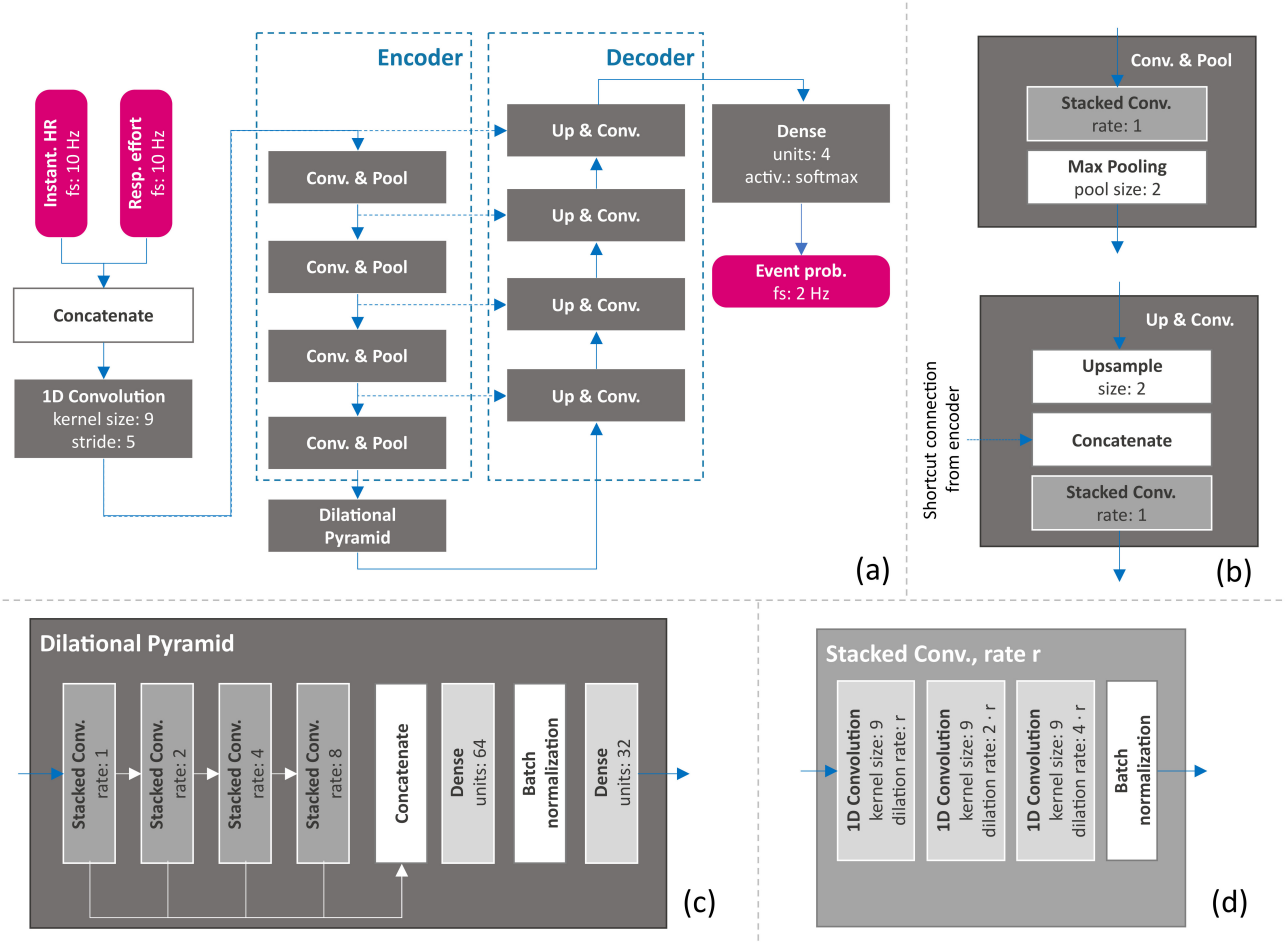
**(a)** Architecture of the neural network, including the encoder-decoder, **(b)** encoder and decoder layers, **(c)** dilational pyramid, and **(d)** each stacked convolutional block. The blocks drawn in red in **(a)** were retrained using accelerometer data from the training part of the present study, while the blocks in gray were kept as pre-trained on ECG and RIP data from a previous study.

The SDB event detection network uses an encoder-decoder architecture with a pyramid pooling block described in earlier work (Figure 5; Fonseca et al., 2024). The encoder represents cardiorespiratory information in feature maps with a progressively lower temporal resolution, while the decoder module up-scales the abstract, low-resolution maps to match the desired temporal output resolution for event detection (2 Hz, same as used in the previous study). A dilational pyramid pooling module combines information at different scales to improve the detection of event boundaries and to help separate events that are temporally close to each other. As shown in the previous description of this neural network, the addition of this block increases the maximum temporal receptive field of the network from 3.8 to 10.8 min, enabling the model to exploit multi-scale contextual information and improving AHI estimation performance.

The network outputs SDB event probabilities, [label **(e)** in Figures 2–4]. Based on the observation that the salience of the expression of SDB events in cardiorespiratory activity depends on the sleep stage in which they occur, we opted to use sleep-stage dependent probability thresholds, optimized as explained in Supplementary material. Using these thresholds, individual events are detected as the group of consecutive output samples where the probability is higher than the threshold defined for the accelerometer-based sleep stage in which they occur [label (f) in Figures 2–4]. These events are subsequently counted and divided by the TST to obtain an estimate of AHI based on accelerometer data (AHI_ACC_).

### 2.4 Training and validation

#### 2.4.1 Sleep staging

The CReSS sleep staging algorithm was used as is, without retraining or in any way tuning to the characteristics of the dataset used in the present study. To evaluate sleep staging performance, we first selected for each recording the interval for which both accelerometer and PSG data were available. Next, TST obtained from chest-wall accelerometry (TST_ACC_) was compared with TST obtained from PSG scoring (TST_PSG_). In addition, for each recording, epoch-per-epoch CReSS sleep stages were compared with the reference sleep stages scored from PSG. Performance was assessed using Cohen's κ of agreement (κ for short) and accuracy for four-class classification (Wake/N1-N2/N3/REM), three-class classification (Wake/NREM/REM; obtained by merging N1-N2 and N3 in a single non-REM or NREM class) and for the binary classification of each of the four classes against the rest (e.g., Wake vs. combined N1, N2, N3, and REM, etc.). For each of the binary classification tasks, sensitivity, specificity, and positive predictive value (PPV) were computed.

#### 2.4.2 Event detection and AHI estimation

To perform transfer learning, we divided the dataset (*N* = 413) into two parts at subject-level, balancing the number of recordings of each OSA severity category between the two. The first part, the training set (*N* = 206), was further randomly split, also at subject-level, into approximately 75% (*N* = 155) of the recordings, which were used for model fitting with transfer learning, and the remaining 25% (*N* = 51) for model selection *via* early stopping to prevent overfitting. The second part, the hold-out set (*N* = 207), was reserved exclusively for evaluating the performance of the adapted AHI estimation algorithm against gold-standard PSG and was completely unseen by any part of the complete system prior to evaluation.

Transfer learning was performed by first initializing all model weights with the values from the pretrained neural network described in the previous study. All 129,998 trainable weights were then iteratively finetuned using the 75% fitting portion of the training set. After each training iteration, we computed the categorical cross-entropy loss on the 25% model selection portion of the training set and used early stopping, terminating training if the loss failed to decrease for 25 consecutive iterations. The final model was defined as the checkpoint with the lowest model selection loss observed during training.

The performance of the AHI estimation algorithm based on accelerometer inputs (AHI_ACC_) was compared against the reference AHI determined from the PSG scorings (AHI_PSG_) using the ICC (two-way random-effects model for absolute agreement) with the corresponding 95% confidence intervals (CIs), and a Bland-Altman analysis of bias and 95% limits of agreement (LoA). Furthermore, we calculated the root mean square error and the mean error (bias) and standard deviation of the error for the complete dataset, as well as for the recordings from each of the two sleep centers.

**Figure 6:**
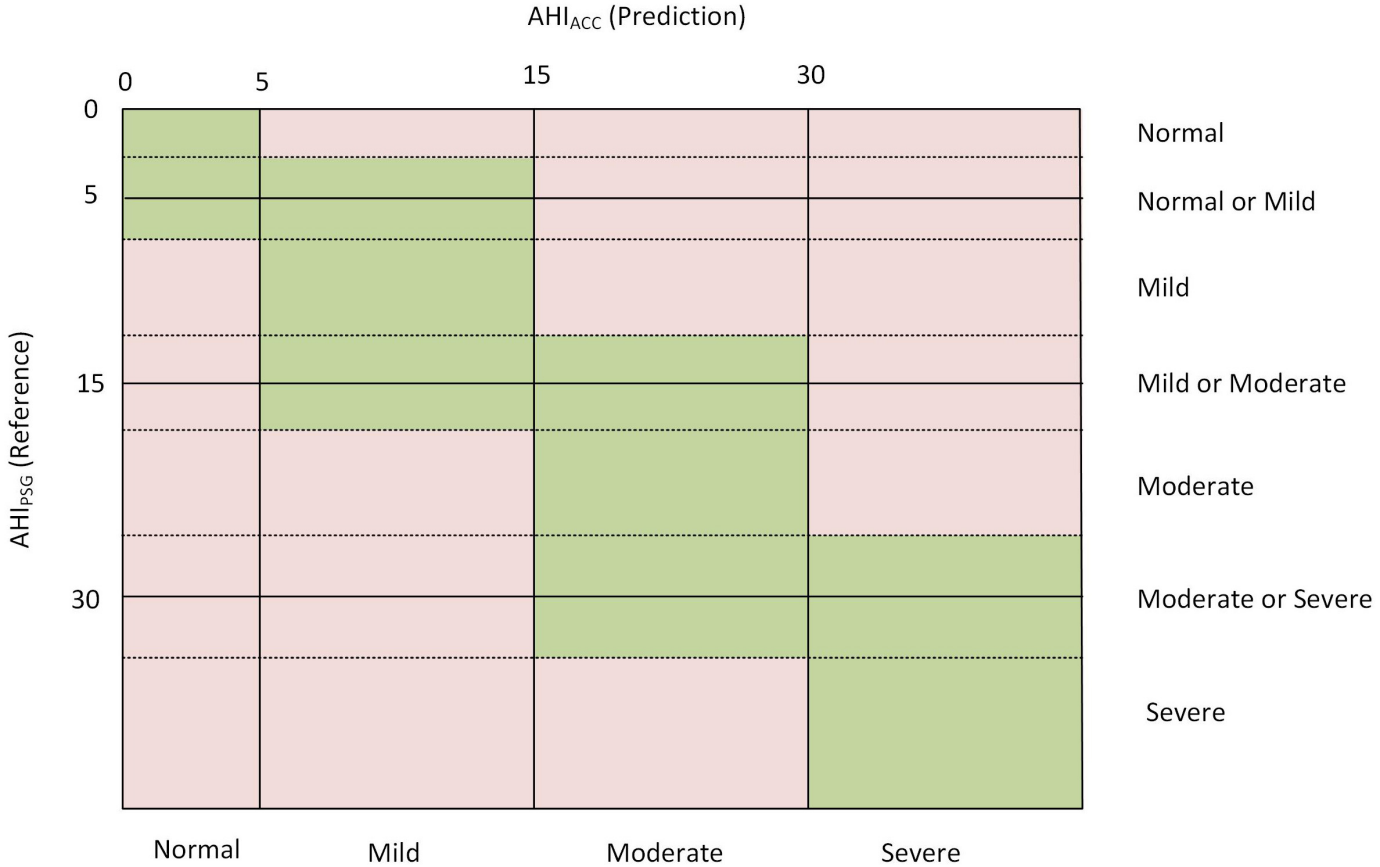
Near boundary double labeling (NBDL). The columns indicate the estimated AHI from acceleration (AHIACC), with the corresponding severity label below the figure; the rows indicate the reference AHI (AHIPSG), with severity label right of the figure. The continuous lines indicate classical boundaries, while the dashed lines indicate NBDL boundaries. In the green areas, prediction and references are considered a match, while in the red areas, prediction and references are considered a mismatch.

The performance in establishing OSA severity was assessed with a four-way confusion matrix from which measures of agreement for the common severity classes “None” (AHI <5), “Mild” (5 ≤ AHI <15), “Moderate” (15 ≤ AHI <30), and “Severe” (AHI ≥ 30) were derived. Furthermore, the algorithm was evaluated in terms of its diagnostic capacity at distinguishing OSA severity at different thresholds, for example, AHI <x vs. AHI ≥ x, with x corresponding to the severity thresholds 5, 15, and 30. Classification performance for each of these binary classification tasks was evaluated using measures of accuracy, sensitivity, specificity, positive and negative predictive values (PPV, NPV), and positive and negative likelihood ratios (LR+, LR-). To account for uncertainty around the canonical thresholds (5, 15, and 30 events/h), and to avoid giving an overly pessimistic view of performance due to small errors around these boundaries, which might not be clinically relevant, we used the near-boundary double-labeling procedure as proposed by Van Pee et al. (2022) and Martinot et al. (2022). Using the boundaries defined by Van Pee and colleagues, we considered the following boundaries: “Normal” (AHI_PSG_ <2.4), “Normal OR Mild” (2.4 ≤ AHI_PSG_ <7.0), “Mild” (7.0 ≤ AHI_PSG_ <12.4), “Mild OR Moderate” (12.4 ≤ AHI_PSG_ <17.4), “Moderate” (17.4 ≤ AHI_PSG_ <26.6), “Moderate OR Severe” (26.6 ≤ AHI_PSG_ <35.2), and “Severe” (AHI_PSG_ ≥ 26.6), in which the zones with “OR” in the label are near-boundary zones, see Figure 6. All reference AHI values were classified into one of these zones. The final confusion matrix was obtained after reducing the near-boundary double-labeled references to a single class out of the standard four severity classes (“None”, “Mild”, “Moderate”, and “Severe”), using the following rules: if the predicted severity matched one of the double-labeled references, the reference was assigned to the row corresponding to the predicted class; otherwise, the reference was assigned to the severity class according to the canonical AHI thresholds (i.e., 5, 15, and 30).

To gain further insight into the potential benefit of transfer learning, we performed two additional comparator experiments. First, we evaluated the original neural network, previously trained on IHR and respiratory effort derived from ECG and RIP, applied directly to accelerometer-derived IHR and respiratory effort inputs without any retraining or weight tuning. Second, we trained a *de novo* model by randomly initializing all weights and fitting the network exclusively on accelerometer-derived inputs. For both experiments, and to enable a direct comparison with transfer learning, we used the identical dataset partitioning described above: the training set (including the internal split for model fitting and model selection) for model development, and the same hold-out set reserved strictly for final evaluation. Performance was assessed with ICC between AHI_ACC_ and AHI_PSG_ in both cases.

#### 2.4.3 Algorithm performance in supine and non-supine positions

The performance of the algorithm was evaluated separately for supine and non-supine body positions. We used the body position sensor, which was part of the PSG, to determine intervals where the participant was lying in supine position and intervals where the participant was lying non-supine (i.e., any other body position except supine). An SDB event that overlapped at least partially with a supine interval during sleep was considered a supine-specific event, otherwise it was considered a non-supine event. Using this rule, we calculated the number of supine and non-supine SDB events detected by the accelerometer, as well as using the scoring from PSG. We also calculated the TST spent in supine and non-supine positions, based on CReSS using accelerometer inputs, and based on the reference scorings from PSG. For all recordings with a minimum of 20 min of TST (according to PSG) in both supine and in non-supine positions, the supine-AHI and non-supine-AHI were calculated for both the PSG and the accelerometer algorithms and evaluated in terms of ICC (two-way random-effects model for absolute agreement).

#### 2.4.4 Statistics

The Shapiro-Wilk normality test was used to determine whether sample data was normally distributed (at a *p*-value of 0.05). Since nearly none of the data analyzed was normally distributed, all sample statistics throughout the manuscript indicate the median, and between curly brackets, the 25th and 75th percentiles: “median {Q1, Q3}” and range between square brackets “[minimum, maximum]”.

To evaluate the potential impact that the accuracy of Acc-IBI and Acc-RE estimation has on the output of the model, we computed the Spearman's r between the absolute AHI estimation error, and separately, the respiratory effort MSE, and the IBI MAE.

## 3 Results

Table 1 presents participant demographics, stratified by participating site and dataset: training (N=206) and hold-out (*N* = 207). SDB was the most common disorder in both datasets (training: 56%; hold-out: 51%), followed by insomnia (25%, 28%), movement disorders (15%, 14%), and REM parasomnia (14%, 13%). A detailed break-down of the prevalence of sleep disorders is available in Supplementary Table S6.

**Table 1:** Demographic information and AHI of participants in the training and hold-out sets.

Site	*N* participants	*N* female (%)	Age (year)	BMI (kg/m^2^)	AHI^*^ (events/h)	TST (h)
Training set (*N* = 206)						
OLVG	68	21 (30.9)	46 {36, 58} [21, 78]	27.0 {24.5, 30.0} [19.0, 39.0]	16.1 {6.3, 27.5} [0.8, 69.4]	7.2 {5.9, 7.8} [1.8, 10.0]
Kempenhaeghe	138	61 (44.2)	52 {34, 62} [13, 83]	26.0 {23.2, 29.3} [18.7, 39.4]	7.0 {3.2, 19.3} [0.0, 78.8]	6.7 {5.5, 7.4} [0.0, 9.0]
Hold-out set (*N* = 207)						
OLVG	60	20 (33.3)	46 {36, 55} [21, 71]	28.0 {25.0, 31.0} [19.0, 47.0]	16.7 {7.3, 27.8} [1.8, 72.8]	6.7 {5.3, 7.5} [2.9, 8.2]
Kempenhaeghe	147	58 (39.5)	53 {38, 64} [17, 80]	25.4 {23.4, 28.7} [17.8, 42.2]	7.5 {2.8, 17.9} [0.2, 72.2]	6.8 {5.5, 7.5} [0.1, 8.5]

BMI, body mass index; AHI, apnea-hypopnea index; TST, total sleep time. For age, BMI, AHI and TST the table indicates the median, between curly brackets, the 25^th^ and 75^th^ percentiles, and between square brackets, the range. ^*^AHI according to PSG.

### 3.1 Demographics

Table 1 presents participant demographics, stratified by participating site and dataset: training (N=206) and hold-out (*N* = 207). SDB was the most common disorder in both datasets (training: 56%; hold-out: 51%), followed by insomnia (25%, 28%), movement disorders (15%, 14%), and REM parasomnia (14%, 13%). A detailed break-down of the prevalence of sleep disorders is available in Supplementary Table S6.

### 3.2 Sleep staging

Comparison of sleep stages derived from chest-wall accelerometry with those derived from PSG yielded a κ-coefficient of agreement of 0.665 for four-class sleep staging and of 0.713 for Wake vs. Sleep classification, see Supplementary Table S1 in the Supplementary material. Using the agreement thresholds from Landis and Koch for interpreting κ, agreement with PSG was substantial for all classification tasks (Landis and Koch, 1977). The best performing class was REM, with a sensitivity of 76.7%, a specificity of 96.7% and a positive predictive value of 81.4%. Results for the recordings of the training set are given in Supplementary Table S2 of the Supplementary material.

The median, first, and third quartiles of the error in TST were +2.5, −9.24, and +14.6 min for the hold-out set, and −1.43, −12.5, and +12.2 min for the training set. Supplementary Figure S2 in Supplementary material illustrates the scatter and Bland-Altman plots for TST estimation for the hold-out set, where the algorithm achieved an ICC of 0.942, with a 95% CI of 0.920 to 0.960 (*p* < 0.001).

### 3.3 AHI estimation

The model was fitted for a total of 16 iterations, after which the loss evaluated on the model selection portion of the training set stopped decreasing. In accordance with the pre-specified early stopping policy, we selected this checkpoint as the final model.

**Figure 7:**
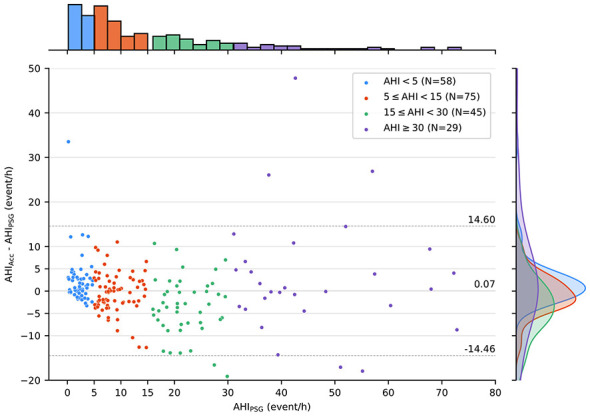
Scatter plot of error per severity class (according to PSG). The histogram above the scatterplot shows the distribution of recordings per severity and the density plot at the right of the scatterplot shows the errors per severity. The solid horizontal line indicates the overall AHI estimation bias, and the dashed lines show the 95% limits of agreement.

Table 2 indicates the performance in AHI estimation on the hold-out set. The algorithm achieved an overall ICC of 0.90, with a bias of −0.07 events/h. The ICC for female (*N* = 78) and male (*N* = 129) was 0.893 and 0.891, respectively, not significantly different (Mann-Whitney U test, *p* = 0.73). Pearson's correlation coefficient reached a value of 0.82 on the AHI against PSG. Figure 7 illustrates the estimation error (AHI_ACC_ minus AHI_PSG_) for each recording in the hold-out set, separately colored per OSA severity according to AHI_PSG_. The algorithm achieved a negligible bias of −0.07 events/h, with 95% LoA of −14.60 and +14.46 events/h. The error distribution is narrowest at AHI_PSG_ <5 and becomes wider with increasing severity. Traditional scatter and Bland-Altman plots are illustrated in Supplementary Figure S3 in the Supplementary material.

**Table 2:** AHI estimation performance for hold-out set, overall and per center.

Center	*N*	*r*	*p*-value (*r*)	ICC	95%CI (ICC)	*p*-value (ICC)	RMSE	Bias	SD error
All	207	0.82	<0.001	0.90	[0.86, 0.92]	<0.001	7.39	−0.07	7.41
Performance per center									
OLVG	60	0.85	<0.001	0.85	[0.76, 0.91]	<0.001	9.30	−0.05	9.38
Kempenhaeghe	147	0.78	<0.001	0.91	[0.88, 0.94]	<0.001	6.45	−0.08	6.47

N, number of recordings for each performance evaluation (“overall” corresponds to the complete hold-out set), r, Spearman's r; ICC, intraclass correlation coefficient for two-way random-effects model for absolute agreement, 95% CI, 95% confidence interval for the ICC; RMSE, root mean square error; SD error, standard deviation of the estimation error.

Table 3 indicates the confusion matrix for OSA severity classification on the hold-out set (N=207), using near-boundary double labeling. The predicted severities matched the reference for 171 recordings, with the predicted severity class off by one class for 32 recordings, by more than one class for only 4 recordings. The four-way κ-coefficient of agreement was 0.76 and the accuracy was 82.6%.

**Table 3:** Confusion matrix for OSA severity classification on the hold-out set using near-boundary double-labeling and after reduction of the reference labels to standard categories.

Ref.↓ Pred. →	No	Mild	Moderate	Severe
No	**54 (81.8%)**	5 (7.2%)	2 (4.5%)	1 (3.6%)
Mild	11 (16.7%)	**54 (78.3%)**	4 (9.1%)	0 (0.0%)
Moderate	1 (1.5%)	10 (14.5%)	**36 (81.8%)**	0 (0.0%)
Severe	0 (0.0%)	0 (0.0%)	2 (4.5%)	**27 (96.4%)**

Each cell indicates the count, and between parenthesis, the distribution of predictions (per column) across the reference severity classes (the diagonal indicates the positive predictive value for the class). The bold values indicate cases where the predicted class matches the reference class.

Table 4 indicates the overall classification performance for each severity threshold, using near-boundary double labeling. Negative likelihood ratios were 0.095, 0.153, and 0.069 using near-boundary double-labeling, and 0.27, 0.23, and 0.14 using classical thresholds for the Mild, Moderate, and Severe thresholds (Supplementary Table S5 in Supplementary material). Positive likelihood rations were 7.1, 16.0, and 165.7 with near-boundary double labeling, and 2.50, 8.16, and 51.15 using classical thresholds for the Mild, Moderate, and Severe thresholds (Supplementary Table S5 in Supplementary material). A breakdown of the rows of Table 4 into the AHI zones as defined in the validation section may be found in Supplementary Table S3 in the Supplementary material. Supplementary Tables S4, S5 in the Supplementary material indicate the confusion matrix and classification performance using classical boundaries.

**Table 4:** Performance for classification at different severity thresholds on the hold-out set, using near-boundary double-labeling.

Min. severity	Prev.≥Sev. (%)	Acc.	Sens.	Spec.	PPV	NPV	LR+	LR-
Mild	70.0	0.903	0.917	0.871	0.943	0.818	7.1	0.095
Moderate	36.7	0.913	0.855	0.947	0.903	0.919	16.0	0.153
Severe	14.0	0.986	0.931	0.994	0.964	0.989	165.7	0.069

Min. severity: minimum severity defined for positive class (i.e., positive class includes this severity and higher) for each binary classification task; Prev.≥Sev.: Prevalence, or percentage of participants with severity equal or greater than the minimum severity; Acc., accuracy; Sens., sensitivity; Spec., specificity; PPV, positive predictive value; NPV, negative predictive value; LR+, positive likelihood ratio; LR-, negative likelihood ratio.

Regarding the comparator experiments, the direct application of the previously trained neural network to accelerometer-derived inputs yielded an ICC of 0.58 (*p* < 0.001, 95% CI: 0.22–0.75), whereas training a new model using only accelerometer-derived features resulted in an ICC of 0.79 (*p* < 0.001, 95% CI: 0.66–s0.87).

Supplementary Tables S7, S8 present the performance of accelerometer derived RE and IBIs in comparison to our previous work. Regarding RE, a significant difference in MSE was observed for the current hold-out set—but not the training set—when compared to earlier results. The Spearman's r between the overall subject-level MSE in RE and the absolute AHI estimation error revealed a weak but significant correlation of 0.30 (*p* < 0.001). Although for the IBIs we encountered significantly higher errors in the current cohort than in the previous work, these errors were extremely small, with subject-level medians being less than 1.4 ms. The Spearman's r between the overall subject-level MAE and the absolute AHI estimation error revealed a weak correlation of 0.14 (*p* = 0.046).

There were four cases where the absolute difference between the estimated AHI (AHI_ACC_) and the reference AHI (AHIP_SG_) was larger than 20 events/h, see Figure 7. Common to these cases was that the AHI was overestimated (AHI_ACC_ > AHI_PSG_). For three of these participants the reference AHI was in the severe class (37, 43, and 57 events/h respectively), with two of them showing a relatively poor performance on IBI (MAE 2.2 and 8.6 IQRs above Q3, respectively, see Supplementary Table S8), and respiratory effort estimation (RMS error 3.9 and 3.8 IQRs above Q3, see Supplementary Table S7). For the remaining participant, the reference AHI was only 0 events/h, with a reference TST of only 4 min, while the present method estimated an AHI of 34 events/h and a TST of 182 min.

### 3.4 Algorithm performance in supine and non-supine positions

Table 5 compares the TST and AHI obtained from PSG and from the accelerometer overall, and per body position (supine, and combination of all non-supine positions), together with the ICC between the PSG and accelerometer-based estimations, for all recordings with a minimum of 20 min on supine and on non-supine positions. For the AHI, an overall ICC of 0.94, with an ICC of 0.88 in the supine position and of 0.93 in the non-supine position. The overall TST reached an ICC of 0.95. Both the overall AHI and TST estimation performances are slightly different than the values reported earlier in this section for the complete hold-out set (*N* = 207), because here we only consider recordings with at least 20 min in supine and non-supine positions (*N* = 147). ICC values for separate body positions can be higher than the overall ICC value, because when calculating the ICC, data is first centered and scaled based on its mean and standard deviation. This eliminates the different biases obtained in TST estimation for each separate body position which led to lower agreement when computing the statistic for both positions combined.

**Table 5:** Comparison between PSG- and accelerometer-derived TST and AHI for the supine and non-supine body positions, on the subset of 147 recordings with a minimum of 20 min of total sleep time (PSG) for each body position.

Body position	TST_PSG_ (h)	TST_Acc_ (h)	ICC_TST_ [95% CI] (-)	Bias [LoA] (h)	AHI_PSG_ (events/h)	AHI_ACC_ (events/h)	ICC_AHI_ [95% CI] (-)	Bias [LoA] (events/h)
All	6.93 {5.90, 7.55}	6.92 {6.06, 7.58}	0.95 [0.93 0.96]	0.06 [−0.79, 0.90]	10.1 {5.29, 21.6}	9.76 {4.45, 17.6}	0.94 [0.92 0.96]	−1.23 [10.15, −12.61]
Supine	2.41 {1.35, 3.45}	2.32 {1.52, 3.47}	0.99 [0.99 0.99]	0.04 [−0.36, 0.44]	18.1 {8.36, 39.6}	14.6 {4.20, 37.3}	0.88 [0.83 0.91]	−2.92 [19.56, −25.41]
Non-supine	3.84 {2.77, 5.29}	4.01 {2.89, 5.42}	0.98 [0.98 0.99]	0.01 [−0.57, 0.60]	4.81 {2.78, 14.5}	6.21 {1.98, 12.9}	0.93 [0.91 0.95]	−0.44 [11.04, −11.92]

TST_PSG_: total sleep time (TST), according to scoring from PSG; TST_Acc_: TST derived from CReSS sleep staging based on accelerometer inputs; ICC_TST_: intraclass correlation coefficient for two-way random-effects model for absolute agreement in total sleep time estimation; 95%CI: 95% confidence interval for the (corresponding) ICC; LoA: 95% limits of agreement, after a Bland-Altman analysis of the estimation error; AHI_PSG_: apnea-hypopnea index (AHI) according to scoring from PSG; AHI_ACC_: AHI estimated from the accelerometer-based algorithm; ICC_AHI_: intraclass correlation coefficient for two-way random-effects model for absolute agreement in AHI estimation, curly brackets: the 25th and 75th percentiles.

Supplementary Figure S4 shows the distributions of the AHI (from PSG), for the supine and non-supine positions.

## 4 Discussion

We developed a method for AHI estimation using an accelerometer on the chest, by combining a cardiorespiratory sleep staging algorithm with an adapted neural network for detecting respiratory events. Performance was evaluated on a hold-out set of 207 participants. Total sleep time was estimated accurately with first and third error quartiles of −9.2 and +15 min, as a basis for accurate AHI estimates. Compared with the PSG-based AHI standard, our chest-wall accelerometer-based model achieved an ICC of 0.90 (95% CI: 0.86–0.92), a negligible bias of 0.07 events/h, and 95% LoA of −14.46 and +14.60 events/h. Using near-boundary double labeling, OSA severity classification reached a Cohen's κ coefficient of agreement of 0.76, indicating substantial agreement with severity classes determined from PSG (Landis and Koch, 1977). The performance of the AHI estimation changed very little if the evaluation intervals were restricted to either supine or non-supine positions. In non-supine position, the algorithm achieved an ICC of 0.93 (95% CI: 0.91–0.95). This is important in the application of PT, as the goal is to have the patient sleep as long as possible in a non-supine position, and thus, the estimation of residual AHI should provide reliable results in this situation. The findings indicate that the method may be suitable for AHI estimation during PT.

Among the three modeling strategies, transfer learning yielded the highest performance (ICC = 0.90), followed by the *de novo* model, trained using only accelerometer-derived inputs (ICC = 0.79), while the direct application of the previously trained ECG/RIP model to accelerometer-based inputs achieved the lowest performance (ICC = 0.58). The poor performance of the direct application indicates that, despite the reasonably accurate estimation of intermediate signals (IHR and RE) from the accelerometer, the neural network remains sensitive to the mismatch between sensing modalities. Training the model from scratch with accelerometer-derived inputs naturally addressed the domain-shift issue, but performance was likely constrained by the relatively limited training dataset (155 recordings, compared with over 2,000 in the original work). In contrast, transfer learning effectively leveraged the pretrained network while adapting the weights to better represent the new input domain. The learning curves shown in Supplementary Figure S1 suggest that early stopping effectively mitigated overfitting. After reaching a minimum loss on the model-selection portion of the training set at iteration 16, the model selection loss progressively increased over the subsequent iterations, consistent with a divergence between training and validation performance suggestive of overfitting. Using as final model the checkpoint with the lowest model selection loss likely minimized the impact of this phenomenon, achieving performance comparable to the original model based on ECG and RIP.

While the present study focused on transferring a previously validated architecture (Fonseca et al., 2024) to accelerometer-derived inputs (Schipper et al., 2023, 2024b), future work could explore alternative architectural components—such as attention mechanisms—which may further improve performance. The output resolution of 2 Hz was retained from the original architecture. While comparable resolutions (e.g., 1–4 Hz) would likely behave similarly, substantially lower resolutions may hinder the separate detection of closely spaced events. Future work may explore these design variants in larger accelerometer datasets.

Regarding the performance of the accelerometer-based cardiorespiratory measurements, a comparison with reference RIP-based respiratory effort and ECG-based IBI showed that the estimation error was relatively low, with significant, but small differences with that obtained in the validation studies ACC-RE-72 (Schipper et al., 2023) and ACC-IBI-147 (Schipper et al., 2024b). However, there was only a weak correlation between the subject-level estimation error estimating IBI and RE (*r* = 0.14, and *r* = 0.30, respectively) and the absolute AHI estimation error, indicating that while signal quality contributes to error, it does not primarily determine the AHI estimation performance of our algorithm.

Regarding cases with a large difference between the estimated and reference AHI, their relatively poor IBI and respiratory effort estimation performance may have played a role. This matches the weak but significant correlation between estimation performance and AHI error that we found, suggesting that in some, but not all cases, the frequent occurrence of SDB events might have negatively impacted the estimation of these parameters, and in turn, degraded the AHI estimation performance. In addition, we found that one of the participants with a high AHI estimation error may have had atrial fibrillation, as visual inspection of the ECG signal revealed irregular R-R intervals and the absence of a P-wave, both indicative of atrial fibrillation. Another participant was diagnosed with Periodic Limb Movement Disorder, which may have disturbed the cardiac and respiratory components in the acceleration signal. Finally, one participant had an extremely low TST, close to 0, whereas the predicted total sleep time was considerably higher, directly contributing to a mismatch in the calculation of the AHI.

Estimating RE and IHR from a chest-worn accelerometer requires a sufficiently low noise floor, as the amplitudes of respiratory and cardiac acceleration components can be very low [see our previous work (Schipper et al., 2023, 2024b) for details]. Furthermore, as cardiac oscillations are mainly visible in relatively high frequency bands, a sufficient bandwidth is required. All data in this study were acquired with the same accelerometer (ADXL355) and with the same sampling rate (250 Hz). In future studies, a sensitivity analysis should be performed to determine the relation between the accelerometer characteristics (such as the noise floor and bandwidth) and the overall performance.

In comparison with currently available methods for AHI estimation during OSA therapy, a recent meta-study of the accuracy of the AHI during PAP therapy by commercially available machines showed 95% LoAs ranging from −15.8 and −4.0, to 0.1 and 18.8 events/h, when evaluated over 12 studies (Iftikhar and BaHammam, 2023). Four of the studies reported ICC values of 0.69 (Nigro et al., 2015), 0.74 (Baek et al., 2016), 0.79 (Berry et al., 2012), and 0.96 (Gagnadoux et al., 2017). In a recent study of 300 OSA patients on PAP therapy (Robbins et al., 2024), it was found that the negative likelihood ratio for AHI estimation was higher than 0.5 for all boundaries and considered by the authors as inconclusive (LR- was 0.58, 0.58, and 0.59 for the boundaries 5, 10, and 15 events/h, respectively). In the present study, we found LR- values of 0.27, 0.23, and 0.14 for the (classical) boundaries 5, 15, and 30 events/h, respectively.

The ICC, the 95% LoA, and the negative likelihood ratio of our method are well within the range of performances reported for PAP machines. This suggests that our method could be equally suitable for AHI monitoring during OSA therapy. However, it is relevant to remark that our participants were measured during diagnostic PSG, whereas the PAP studies evaluated participants during therapy. Therefore, range of the AHI values may vary between the two populations, with lower AHI values observed during PAP therapy and higher values in our study.

In comparison with other work using inertial sensors, our results align with the diagnostic accuracy observed in a study using mandibular jaw movements, from which a median bias of 0.24 events/h and LoAs of −11.2 to +12.8 events/h (Pepin et al., 2022). Although they did not focus on the estimation of AHI, Hsu et al. demonstrated that a ‘chest-effort index' based on exertion of chest-wall movements could classify moderate-to-severe OSA (AHI ≥ 15) with 80% sensitivity and 79.4% specificity (Hsu et al., 2020). Ryser et al. (2022) reported a sensitivity of 76% and specificity of 70% for the detection of “breathing cessations” using the magnitude of chest-wall movements (Ryser et al., 2022). Our reported sensitivity of 85.5% and specificity of 94.7% suggest that high-fidelity cardiorespiratory measurements (respiratory effort and instantaneous heart rate) may provide a more robust assessment of OSA than indices derived solely from magnitude or exertion. Nonetheless, this interpretation should be viewed with some caution, until independent replication studies and -ideally- head-to-head comparisons become available.

Regarding the potential use during PT, it should be noted that of course performance in different body positions in this study was evaluated without any form of therapeutic intervention. During actual positional therapy, the stimulation used to trigger the patient to change from a supine to a non-supine position might influence sleep architecture and perhaps introduce artifacts that could negatively impact AHI estimation performance. This effect may be limited however, given the relatively small number of positional changes during a night, compared to for example the number of naturally occurring arousals, or even residual apnea and hypopnea events. AHI estimation performance may in practice also be impacted by the accelerometer placement and body contact; here the devices were mounted on a respiratory thoracic RIP belt by professional staff, while during therapy the devices are mounted by the patients themselves.

In the context of a diagnostic application, the method demonstrated relatively high positive likelihood ratios for AHI severity classification (2.50, 8.16, and 51.1 for the Mild, Moderate, and Severe classes, respectively, using classical boundaries). For comparison, in a recent meta-study on peripheral arterial tonometry (PAT; Iftikhar et al., 2022), positive likelihood ratios of 1.66, 3.34, and 5.74 were found for the same boundaries. A few studies on PAT reported an ICC for the AHI, over cohorts with varying characteristics. In one study, among 30 participants suspected of having OSA, an ICC of 0.88 was found when comparing against PSG (Pittman et al., 2004). In an early PAT meta study, a Pearson correlation of 0.89 was found (Yalamanchali et al., 2013) when comparing the AHI against that derived from PSG, while the overall value found in the present study was 0.82.

Although not fully comparable with previous studies because of differences in study cohorts and in scoring parameters (for example 3 vs. 4% desaturation in case of hypopneas), the relatively high positive likelihood ratios found in this study indicate that the method might be usable for diagnostic purposes. An interesting practical use case may be to integrate the method in wearable patches for extended Holter ECG monitoring. In this case, the IHR may be derived from the ECG signal, while respiration can be derived from an added accelerometer (Janbakhshi and Shamsollahi, 2018). Since OSA is common in patients with cardiac disorders including arrythmias population, with prevalences reported up to 74% (Linz et al., 2022) and the burden of OSA may worsen the cardiac condition (Azarbarzin et al., 2020), there is clinical value in having an estimation of possible OSA severity for further follow up (Linz et al., 2018).

Our method might also be useful as a backup sensing modality during sleep recordings, for example in type II sleep tests (unsupervised polysomnography). These tests require a high number of sensors that need proper galvanic or optical skin contact, and poor recording quality is not uncommon. Using alternative signals from an accelerometer device may save otherwise unusable recordings. Provided that the accelerometer has adequate performance, these signals could then be processed by the algorithms presented here and in our previous work (Schipper et al., 2023, 2024a,b), as indicated in Figure 2. This would allow the estimation of not only the AHI, but also of a simplified, 4-class hypnogram, differentiating Wake, REM, N1/N2, and N3. These sleep stages can then be used to compute classical, overnight sleep statistics reflecting sleep and wake (e.g., sleep onset latency, sleep efficiency, etc.), and sleep stage distributions (e.g., percentage of N3, REM, etc.).

A limitation of this study was that we could not evaluate the performance in the presence of cardiac rhythm disorders. Patients with a documented history of cardiac arrhythmias (e.g., atrial fibrillation) were not included. For the participants included, we did not inspect the recordings for possible arrhythmia and, therefore, did not exclude any recordings based on this assessment. However, it is plausible to assume that in the presence of arrhythmia, the cardiac measurements themselves may be less reliable and the relationship between these measurements and sleep apnea may have been insufficiently captured in the present dataset. This should be investigated in future studies.

Furthermore, the proposed method is not capable of distinguishing obstructive from central apnea events. In future research, it could be investigated if full absence of respiratory movements could be used to discern central from obstructive events. Furthermore, the method was evaluated among patients undergoing polysomnography and the sensors were mounted by clinical staff. In future studies, the method should be evaluated in application scenarios, possibly with self-attachment of the sensor and during actual OSA therapy, in particular PT, which currently lacks any measurement of AHI or sleep.

In summary, this study demonstrates the potential of accurately measuring AHI using chest-worn accelerometry, achieving substantial agreement with PSG-derived AHI. Importantly, accelerometer-based AHI estimation performance remained consistent across all body positions, and comparable to the performance of residual AHI measurement during PAP therapy. This method may be of value in various clinical applications, in particular in the treatment of OSA with PT. By offering the possibility of measuring sleep stages and AHI on a nightly basis for patients undergoing this therapy, it enriches the currently available treatment information which consists simply of usage time, and a sleep position summary. As the severity of the disorder may vary or worsen over time, and eventually even manifest itself in non-supine positions, continuous monitoring of sleep and AHI during treatment may substantially enhance the assessment of therapy efficacy and the management of the disorder.

## Data Availability

The datasets presented in this article are not readily available because “Please check with the corresponding author on which data are available”. Requests to access the datasets should be directed to overeems@kempenhaeghe.
