# Supplementary material for: Apnea-hypopnea index estimation using overnight chest-wall accelerometry
Source: Front Sleep. 2026 Jul 20;5:1858267. doi: 10.3389/frsle.2026.1858267 (PMC13429486; doi:10.3389/frsle.2026.1858267)
Supplement: Supplementary file 1 [file Supplementary_file_1.docx]

Supplementary Materials to “Apnea-Hypopnea Index estimation using overnight chest-wall accelerometry”

*Schipper, F.*^,1,2^, Fonseca, P.*^,1,2^, Grassi, A.^1^, Ross,M.^2,3^, van Meulen, F.B.^2,4^, van Gilst, M.^2,4^, Bosschieter, P.F.N.^2,4^, Schoustra, E.^5^, van Sloun, R.J.G. ^2^, Abdenbi, F.^1^, de Vries, N.^5^, Heinzer, R.^6^, Pépin, J.L.^7^, Overeem,S.^2,4^*

**These authors contributed equally to this work.*

^1^Philips Sleep and Respiratory Care, Eindhoven, the Netherlands

^2^Department of Electrical Engineering, Eindhoven University of Technology, Eindhoven, the Netherlands

^3^The Siesta Group, Vienna, Austria

^4^Center for Sleep Medicine Kempenhaeghe, Heeze, the Netherlands

^5^Department of Otorhinolaryngology, Head and Neck Surgery OLVG West, Amsterdam, the Netherlands

^6^Centre d'investigation et de recherche sur le sommeil, Lausanne, Switzerland

^7^Université Grenoble Alpes: Grenoble, Rhône-Alpes, France

# Neural network training

Transfer learning was performed by further fitting the neural network, pre-trained using ECG and respiratory effort inputs as described in a previous study [1], to the characteristics of the accelerometer inputs. Concretely, the network was adapted using the IHR and modified respiratory effort estimated from the accelerometer signals of the training set, using a similar training procedure as with the original study [1]. We used an Adam optimizer, categorical cross-entropy loss, and an early stopping strategy, interrupting training after the validation loss (evaluated on 25% of the recordings of the training set) stopped improving for 25 training iterations.

Figure S1 illustrates the learning curves for the transfer learning procedure. The “fitting” loss was computed on the 75% portion of the training set used for parameter optimization, while the (model) “selection” loss was evaluated on the remaining 25% used for early stopping. The model was trained for a total of 41 iterations, with the lowest model selection loss occurring at iteration 16, after which it no longer decreased. The final model parameters were therefore taken from this iteration. Training required 2 minutes and 14.6 seconds on an NVIDIA GeForce GTX 1080 Ti GPU. The minimum model selection loss was reached after 55.9 seconds (iteration 16).


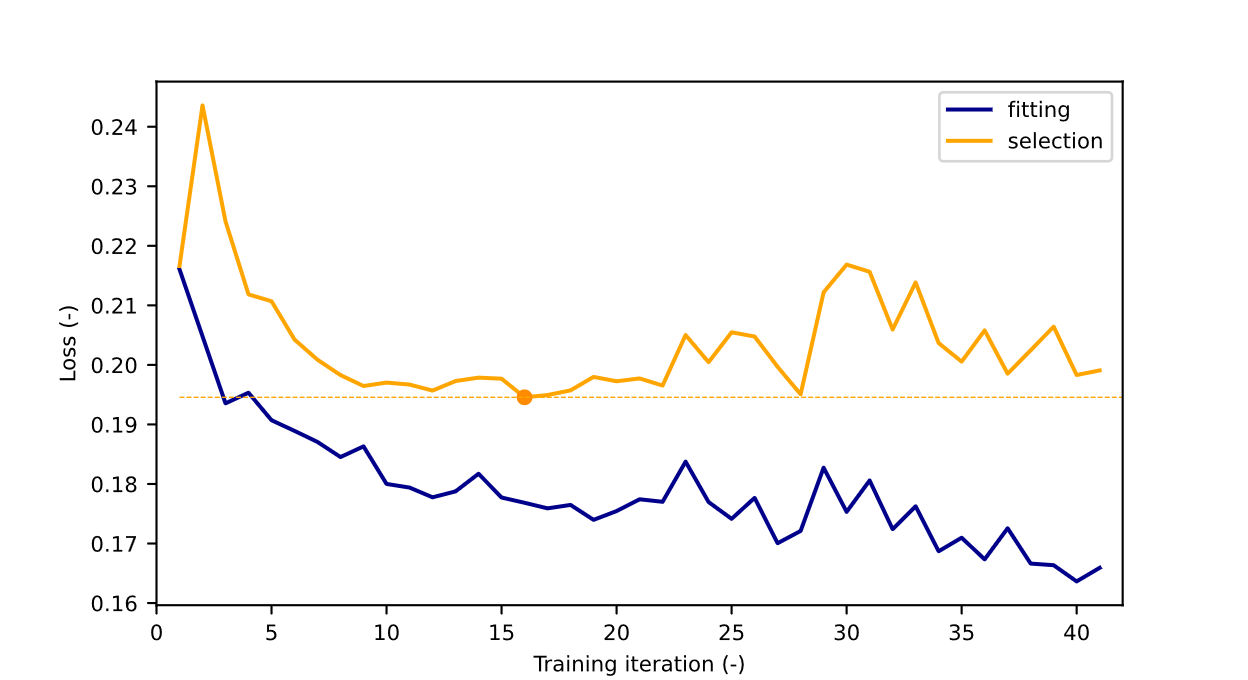


Figure S1 – learning curves for loss evaluated on the (model) fitting and on the (model) selection portions of the training set and checkpoint iteration (indicated with a dot) used for the final model.

# Sleep stage-specific probability thresholds

The original neural network for event detection [1] uses estimated sleep stages as inputs. In this way, the network could deal with differences in how apneas and hypopneas manifest themselves in the respiratory effort and instantaneous heart rate signals, in different sleep stages. In the present study we opted for a different approach, as outlined in the top part of Figure 2. Instead of using sleep stages as inputs to the neural network, we used them to determine sleep stage-specific thresholds which were in turn used to detect apnea and hypopnea events (label (f) in Figures 1 and 2). In contrast with the previous study, where a single probability threshold was used for all sleep stages, in the present study the probability outputs were compared against the threshold optimized (based on the training data) for the sleep stage where each output sample occurs.

The network outputs separate probabilities for obstructive apnea, central apnea, and hypopnea (label (f) in Figures 1 and 2). To predict which output samples belong to an SDB event, we calculated the maximum across these probabilities and used sleep stage-specific thresholds to determine sequences of consecutive output samples where the maximum probability exceeds the threshold for the corresponding sleep stage detected by the cardiorespiratory sleep staging algorithm (CReSS) used in the study [2] (label (f) in Figure 2). Probabilities (of any value) occurring during (detected) Wake were ignored. Uninterrupted sequences of consecutive output samples above the threshold were counted as individual SDB events. AHI was computed as the number of detected SDB events, divided by the total sleep time estimated from sleep staging. The sleep-stage specific thresholds were automatically determined using grid search as the set of thresholds that maximized the intraclass correlation coefficient (ICC; two-way random-effects model for absolute agreement) between the resulting AHI estimation based on accelerometer inputs (AHI_ACC_) and the AHI determined from PSG (AHI_PSG_) on the recordings of the training set.

# Sleep staging

Table S1 shows the sleep staging performance obtained with the pre-trained cardiorespiratory sleep staging algorithm (CReSS) [2] on the hold-out set, while Table S1 shows the sleep staging performance on the training set. It should be emphasized that none of these recordings were used to retrain or tune in any way the sleep staging algorithm, which was used as is (here the nomenclature ‘training’ refers to the fact that these recordings were used to train the SDB event detection algorithm).

Table S1 – Sleep staging performance for different classification tasks, on the hold-out set (N=207).

| **Task** | **κ (-)** | **Accuracy (%)** | **Sens. (%)** | **Spec. (%)** | **PPV (%)** |
| --- | --- | --- | --- | --- | --- |
| Wake/N1-N2/N3/REM | 0.665 {0.575, 0.750} | 80.3 {75.5, 85.0} | n/a | n/a | n/a |
| Wake/NREM/REM | 0.737 {0.639, 0.805} | 87.3 {83.1, 90.5} | n/a | n/a | n/a |
| N1-N2 | 0.612 {0.510, 0.696} | 81.3 {77.1, 85.5} | 85.1 {79.5, 90.5} | 79.0 {70.8, 84.7} | 82.4 {76.7, 88.7} |
| N3 | 0.612 {0.359, 0.773} | 93.5 {90.8, 95.8} | 71.4 {50.4, 86.5} | 97.7 {94.7, 99.4} | 76.2 {47.4, 92.3} |
| REM | 0.782 {0.632, 0.867} | 95.0 {92.1, 96.9} | 84.6 {70.1, 91.5} | 97.8 {95.8, 99.1} | 86.7 {69.7, 94.6} |
| Wake (vs. Sleep) | 0.713 {0.621, 0.790} | 93.1 {89.6, 95.3} | 76.7 {64.6, 87.3} | 96.7 {93.7, 98.1} | 81.4 {67.9, 90.1} |

Acc: accuracy, Sens: sensitivity, Spec: specificity, PPV: positive predictive value, curly brackets: the 25^th^ and 75^th^ percentiles. Binary classification tasks were evaluated in a one vs. rest strategy, where one single class (e.g. Wake, REM, etc) was considered the "positive" class, and the remaining were aggregated in a single "negative" class.

Table S2 – Sleep staging performance for different classification tasks, on the training set (N=206).

| **Task** | **κ (-)** | **Accuracy (%)** | **Sens. (%)** | **Spec. (%)** | **PPV (%)** |
| --- | --- | --- | --- | --- | --- |
| Wake/N1-N2/N3/REM | 0.694 {0.610, 0.765} | 81.7 {77.4, 85.9} | n/a | n/a | n/a |
| Wake/NREM/REM | 0.754 {0.670, 0.823} | 88.5 {84.4, 91.2} | n/a | n/a | n/a |
| N1-N2 | 0.635 {0.555, 0.710} | 82.5 {78.6, 86.2} | 86.6 {81.0, 90.5} | 79.3 {71.0, 87.5} | 84.0 {77.3, 89.1} |
| N3 | 0.670 {0.506, 0.777} | 94.0 {92.1, 96.1} | 72.3 {56.3, 87.7} | 97.7 {95.0, 99.4} | 74.9 {56.2, 91.5} |
| REM | 0.804 {0.711, 0.881} | 95.6 {92.7, 97.1} | 84.1 {70.5, 90.9} | 98.1 {96.2, 99.2} | 87.9 {76.4, 94.6} |
| Wake (vs. Sleep) | 0.706 {0.590, 0.794} | 93.2 {90.1, 95.4} | 80.0 {65.4, 89.8} | 96.3 {94.1, 97.9} | 78.2 {65.9, 88.6} |

Acc: accuracy, Sens: sensitivity, Spec: specificity, PPV: positive predictive value

Note 1: the CReSS algorithm was not trained, or in any way adjusted, based on this portion of the dataset, since it was used as is. ‘training’ refers to the fact that this set was used to train the AHI estimation algorithm, but not CReSS.

Note 2: binary classification tasks were evaluated in a one vs. rest strategy, where one single class (e.g. Wake, REM, etc) was considered the "positive" class, and the remaining were aggregated in a single "negative" class.

Figure S2 shows the scatter and Bland-Altman plots for the total sleep time (TST) on the hold-out set.


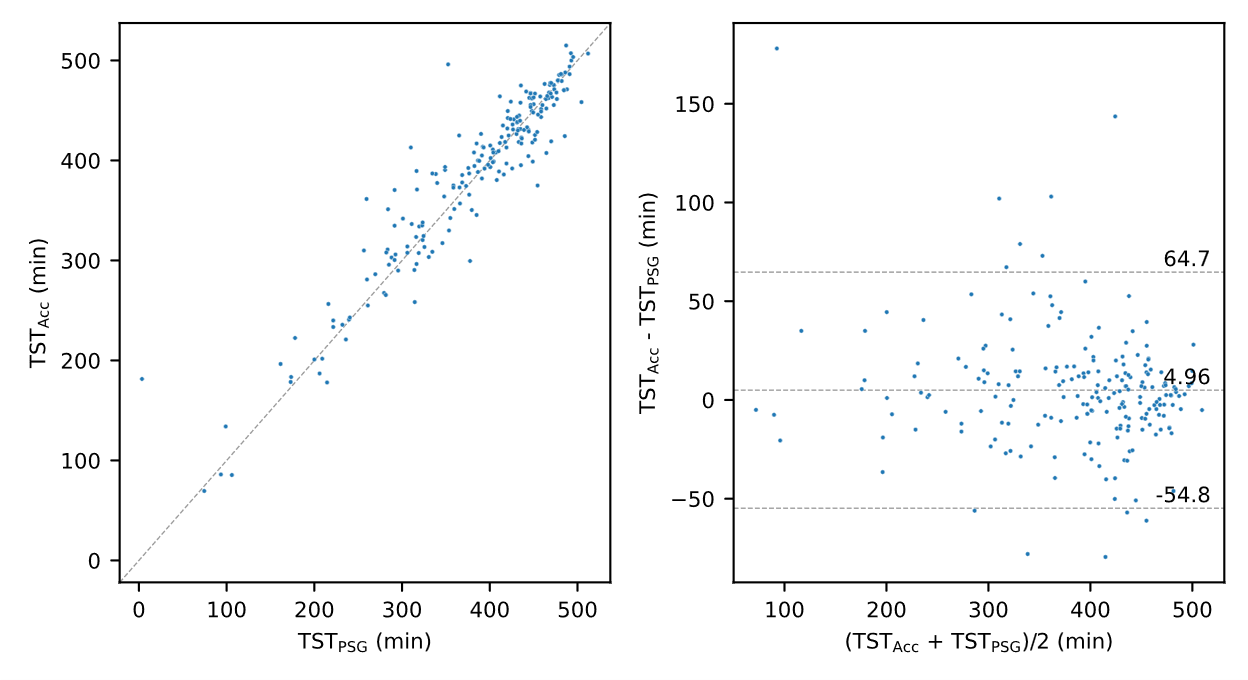


Figure S2 – (left) scatter plot and (right) Bland-Altman plots for estimated TST (TSTAcc) versus reference TST from PSG (TSTPSG) in the hold-out set. The dashed horizontal lines in the Bland-Altman plot indicate the bias (mean of the estimation error) and the 95% limits of agreement in minutes.

# AHI estimation

Figure S3 illustrates the scatter and Bland-Altman plots for AHI estimation in the hold-out set.


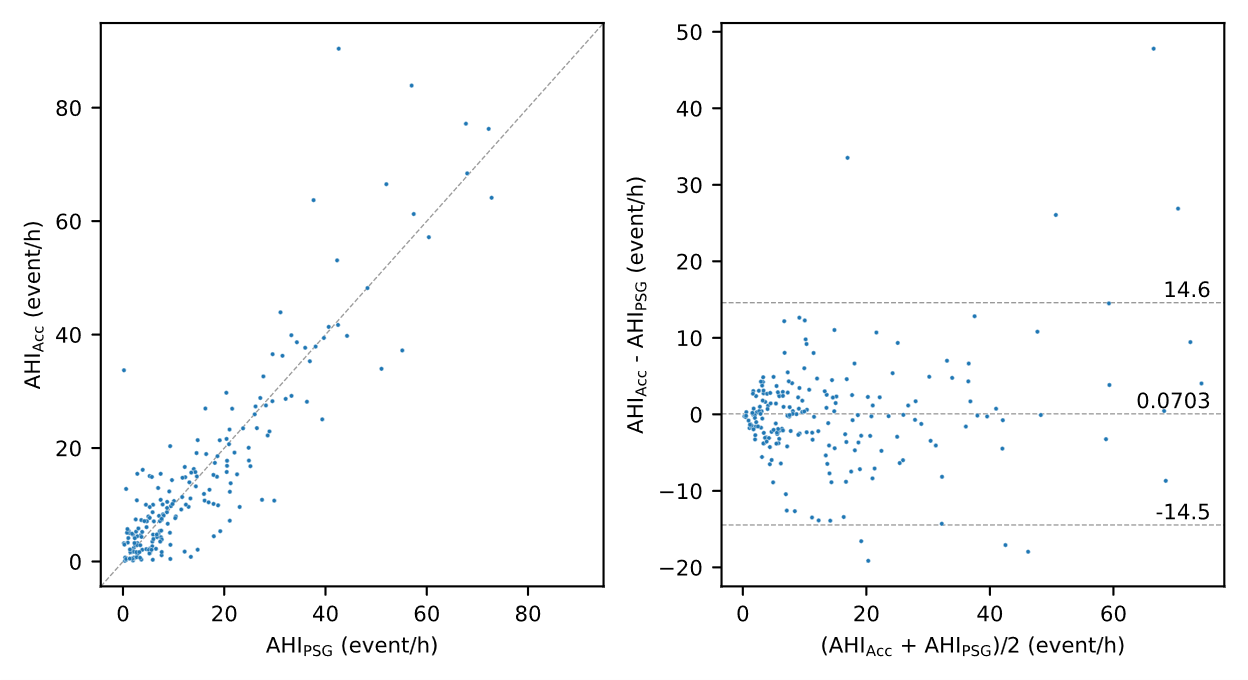


Figure S3 – (left) scatter plot and (right) Bland-Altman plots for estimated AHI (AHIAcc) versus reference AHI from PSG (AHIPSG) in the hold-out set. The dashed horizontal lines in the Bland-Altman plot indicate the bias (mean of the estimation error) and the 95% limits of agreement (bias ± of the estimation error) in events/h standard deviation.

Table S3 gives a breakdown of the rows of the confusion matrix of Table 3 into the near-boundary AHI zones, as defined in the validation section.

Table S3 – Confusion matrix for OSA severity classification on the hold-out set using near-boundary double-labeling for the reference severity.

|  | **Pred.→** | **None** | **Mild** | **Moderate** | **Severe** |
| --- | --- | --- | --- | --- | --- |
| **Ref.(NBDL)↓** |  | **[0, 5)** | **[5, 15)** | **[15, 30)** | **≥30** |
| **None** | **[0.0, 2.4)** | 24 | 5 | 0 | 1 |
| **None or mild** | **[2.4, 7.0)** | 30 | 19 | 3 | 0 |
| **Mild** | **[7.0, 12.4)** | 9 | 26 | 3 | 0 |
| **Mild or moderate** | **[12.4, 17.4)** | 2 | 9 | 8 | 0 |
| **Moderate** | **[17.4, 26.6)** | 1 | 8 | 21 | 0 |
| **Moderate or severe** | **[26.6, 35.2)** | 0 | 2 | 7 | 6 |
| **Severe** | **≥35.2** | 0 | 0 | 2 | 21 |

Ref.(NBDL): reference class, according to PSG, using near-boundary double-labeling, Pred.: predicted class, based on the predicted AHI, AHI_Acc._ The intervals next to and below the severity classes indicate the AHI values for each severity category, with [x, y) indicating the AHI interval corresponding to that severity category, i.e., x ≤ AHI < y_._

Percentages between parenthesis indicate the percentage of each reference class relative to the total number of predictions of that class.

Light gray cells indicate classifications that are considered correct using near-boundary double-labeling.

Tables S4 and S5 indicate the confusion matrix and classification performance of AHI estimation using classical severity boundaries.

Table S4 - Confusion matrix for OSA severity classification on the hold-out set, using the classical boundaries.

|  | **Pred. →** | **No** | **Mild** | **Moderate** | **Severe** |
| --- | --- | --- | --- | --- | --- |
| **Ref.↓** |  | **[0, 5)** | **[5, 15)** | **[15, 30)** | **≥30** |
| **No** | **[0, 5)** | **39 (59.1%)** | 16 (23.2%) | 2 (4.5%) | 1 (3.6%) |
| **Mild** | **[5, 15)** | 26 (39.4%) | **39 (56.5%)** | 10 (22.7%) | 0 (0.0%) |
| **Moderate** | **[15, 30)** | 1 (1.5%) | 14 (20.3%) | **28 (63.6%)** | 2 (7.1%) |
| **Severe** | **≥30** | 0 (0.0%) | 0 (0.0%) | 4 (9.1%) | **25 (89.3%)** |

Ref.: reference class, according to PSG, Pred.: predicted class, based on AHI _Acc._

Percentages between parenthesis indicate the percentage of each reference class relative to the total number of predictions of that class (on the diagonal, these percentages correspond to the positive predictive value for the corresponding class).

Table S5 - Performance for classification at different severity thresholds on the hold-out set, using classical boundaries

| **T (events/h)** | **Prev.≥T (%)** | **Acc.** | **Sens.** | **Spec.** | **PPV** | **NPV** | **LR+** | **LR-** |
| --- | --- | --- | --- | --- | --- | --- | --- | --- |
| 5 | 72.0 | 0.778 | 0.819 | 0.672 | 0.865 | 0.591 | 2.499 | 0.269 |
| 15 | 35.7 | 0.865 | 0.797 | 0.902 | 0.819 | 0.889 | 8.157 | 0.225 |
| 30 | 14.0 | 0.966 | 0.862 | 0.983 | 0.893 | 0.978 | 51.149 | 0.140 |

T: AHI threshold used for each binary classification, e.g. AHI>5 versus AHI≤5, etc.; Prev.≥T: Prevalence, or percentage of participants with AHI equal or greater than the threshold; Acc.: accuracy; Sens.: sensitivity; Spec.: specificity; PPV: positive predictive value; NPV: negative predictive value; LR+: positive likelihood ratio; LR-: negative likelihood ratio.

Table S6 gives the prevalence of sleep disorders over the training and hold-out sets.

Table S6 – Prevalence of sleep disorders over the train and hold-out sets.

|  | Training set (N=206) | | Hold-out set (N=207) | |
| --- | --- | --- | --- | --- |
| Disorder | N | % | N | % |
| Sleep disordered breathing | 115 | 56 | 106 | 51 |
| Insomnia | 51 | 25 | 58 | 28 |
| Sleep related movement disorder | 31 | 15 | 30 | 14 |
| REM parasomnia | 29 | 14 | 26 | 12 |
| Central disorder of hypersomnolence | 19 | 9 | 16 | 8 |
| Non-REM parasomnia | 12 | 6 | 11 | 5 |
| Other parasomnia | 11 | 6 | 8 | 4 |
| Circadian | 6 | 3 | 5 | 2 |
| Central sleep apnea | 2 | 1 | 4 | 2 |
| None | 1 | 0 | 3 | 1 |

Note that a participant may have been diagnosed with multiple disorders, which is why the sum of the numbers
in the N-column is larger than the number of participants in the set.

Figure S4 shows the distributions of the AHI (from PSG), for the supine and non-supine positions.


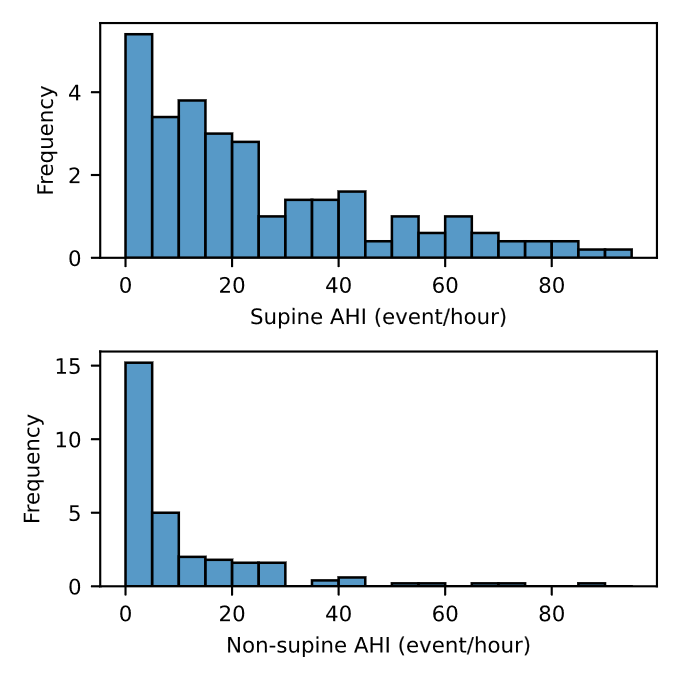


Figure S4 – Distribution of AHI (according to PSG) per body position for the recordings in the hold-out set. For non-supine sleeping positions, the AHI score is frequently low, whereas for supine positions, it is more often high.

# Estimation of respiratory effort and interbeat intervals

To evaluate the performance of accelerometer‑based respiratory‑effort estimation, we followed the same overall procedure as in the previous cohort ACC-RE-72 [3] by computing segment‑level mean squared error (MSE) between the thoracic RIP signal and the accelerometer‑derived estimate of respiratory effort. In the present study, however, we refined the analysis by first calculating, for each participant, the median segment‑level MSE, and only then deriving sample‑level statistics for the full hold‑out set. To ensure a fair comparison across studies, we recomputed the subject‑level MSEs for the cohort of the previous publication using this same procedure. Table S7 reports these subject‑level MSE values for both cohorts.

Table S7 - Subject-level respiratory effort MSE statistics over earlier publication for the training and hold-out sets.

| **Dataset** | **n** | **Median (a.u.)** | **Q1-Q3 (a.u.)** | **vs. earlier study*  (p-value)** | **vs. training set** (p-value)** |
| --- | --- | --- | --- | --- | --- |
| ACC-RE-72 [3] | 72 | 0.108 | 0.053 - 0.198 | - | - |
| Training set | 206 | 0.130 | 0.061 - 0.244 | 0.13 | - |
| Hold-out set | 207 | 0.155 | 0.088 - 0.297 | 0.0023 | 0.041 |

*Mann-Whitney U test for significant differences, between the MSE in the previous study, and in each current study subset; **Mann-Whitney U test for significant differences, between the MSE in the training and hold-out sets of the current study

A similar procedure was used to evaluate the performance of accelerometer‑based IBI estimation. As in the previous cohort ACC-IBI-147 [4], reference IBIs were obtained by detecting R‑peaks in the ECG signal and by computing the time differences between successive beats. For each recording, accelerometer‑detected heartbeats were matched to ECG‑detected heartbeats using their localization times while allowing for a 250 ms tolerance window. The IBI error, defined as the time difference between the accelerometer and ECG IBIs, was then used to compute the mean absolute error (MAE) per beat. These MAE values were subsequently aggregated into subject‑level statistics for both cohorts. Table S8 reports the resulting subject‑level MAE values for IBI estimation.

Table S8 – Subject-level IBI MAE statistics over earlier publication, for the training and hold-out sets.

| **Dataset** | **n** | **Median (ms)** | **Q1-Q3 (ms)** | **vs. earlier study*  (p-value)** | **vs. training set** (p-value)** |
| --- | --- | --- | --- | --- | --- |
| ACC-IBI-147 [4] | 147 | 4.07 | 3.22 - 5.58 | - | - |
| Training set | 206 | 4.95 | 3.57 - 7.40 | < 0.001 | - |
| Hold-out set | 207 | 5.46 | 3.87 - 8.21 | < 0.001 | 0.19 |

*Mann-Whitney U test for significant differences, between the MSE in the previous study, and in each current study subset; **Mann-Whitney U test for significant differences, between the MSE in the training and hold-out sets of the current study

# References

[1] Fonseca P, Ross M, Cerny A, Anderer P, Schipper F, Grassi A, et al. Estimating the severity of obstructive sleep apnea using ECG, respiratory effort and neural networks. IEEE J Biomed Health Inform 2024;28:3895–906. <https://doi.org/10.1109/JBHI.2024.3383240>.

[2] Bakker JP, Ross M, Vasko R, Cerny A, Fonseca P, Jasko J, et al. Estimating sleep stages using cardiorespiratory signals: validation of a novel algorithm across a wide range of sleep-disordered breathing severity. J Clin Sleep Med 2021;17:1343–54. <https://doi.org/10.5664/jcsm.9192>.

[3] Schipper F, JG van Sloun R, Grassi A, Overeem S, Fonseca P. A deep-learning approach to assess respiratory effort with a chest-worn accelerometer during sleep. Biomedical Signal Processing and Control 2023;83:104726. <https://doi.org/10.1016/j.bspc.2023.104726>.

[4] Schipper F, Van Sloun RJG, Grassi A, Brouwer J, Van Meulen F, Overeem S, et al. Maximum a posteriori detection of heartbeats from a chest-worn accelerometer. Physiol Meas 2024;45:035009. <https://doi.org/10.1088/1361-6579/ad2f5e>.
